# Evaluating the comparative effectiveness of different demand side interventions to increase maternal health service utilization and practice of birth spacing in South Kivu, Democratic Republic of Congo: an innovative, mixed methods approach

**DOI:** 10.1186/s12884-017-1396-3

**Published:** 2017-07-03

**Authors:** Mari Dumbaugh, Wyvine Bapolisi, Jennie van de Weerd, Michel Zabiti, Paula Mommers, Ghislain Bisimwa Balaluka, Sonja Merten

**Affiliations:** 1 Swiss Tropical & Public Health Institute, University of Basel, Basel, Switzerland; 2 Ecole Régionale de Santé Publique, Université Catholique de Bukavu, Bukavu, Democratic Republic of Congo; 3 CORDAID-Netherlands, The Hague, Netherlands; 4 CORDAID-Bukavu, Bukavu, Democratic Republic of Congo

**Keywords:** Maternal health, Service utilization, Conditional cash transfer, Demand side financing, Community health workers, Family planning, Mixed methods, Study protocol, Democratic Republic of Congo

## Abstract

**Background:**

In this protocol we describe a mixed methods study in the province of South Kivu, Democratic Republic of Congo evaluating the effectiveness of different demand side strategies to increase maternal health service utilization and the practice of birth spacing. Conditional service subsidization, conditional cash transfers and non-monetary incentives aim to encourage women to use maternal health services and practice birth spacing in two different health districts. Our methodology will comparatively evaluate the effectiveness of different approaches against each other and no intervention.

**Methods/design:**

This study comprises four main research activities: 1) Formative qualitative research to determine feasibility of planned activities and inform development of the quantitative survey; 2) A community-based, longitudinal survey; 3) A retrospective review of health facility records; 4) Qualitative exploration of intervention acceptability and emergent themes through in-depth interviews with program participants, non-participants, their partners and health providers. Female community health workers are engaged as core members of the research team, working in tandem with female survey teams to identify women in the community who meet eligibility criteria. Female community health workers also act as key informants and community entry points during methods design and qualitative exploration. Main study outcomes are completion of antenatal care, institutional delivery, practice of birth spacing, family planning uptake and intervention acceptability in the communities. Qualitative methods also explore decision making around maternal health service use, fertility preference and perceptions of family planning.

**Discussion:**

The innovative mixed methods design allows quantitative data to inform the relationships and phenomena to be explored in qualitative collection. In turn, qualitative findings will be triangulated with quantitative findings. Inspired by the principles of *grounded theor*y, qualitative analysis will begin while data collection is ongoing. This “conversation” between quantitative and qualitative data will result in a more holistic, context-specific exploration and understanding of research topics, including the mechanisms through which the interventions are or are not effective. In addition, engagement of female community health workers as core members of the research team roots research methods in the realities of the community and provides teams with key informants who are simultaneously implicated in the health system, community and target population.

## Background

Increasing women’s access to quality health services forms a cornerstone of many health programs in developing countries. However service utilization along the spectrum of maternal health care, especially in sub-Saharan Africa (SSA), remains low [1, 2]. Numerous recent publications explore barriers to access and utilization of services [3–10] demonstrating that in a variety of contexts these questions remain unanswered and highly relevant.

Demand side health interventions are implemented in different ways and aim to incentivize individuals to access particular health services or adopt certain behaviors [11]. Conditional cash transfers (CCTs) were first implemented in Latin American countries as a kind of “social safety net” – regular cash payments were targeted at poor families who complied with “multiple conditionalities” [12] such as children’s school attendance, participation in health information sessions and medical check-ups [12, 13].In recent years CCTs won the endorsement of the World Bank [14] heralded as solutions to reducing cost barriers to health services by correcting for “market failures” [15]. CCTs are increasingly used in SSA but, unlike their Latin American predecessors, usually do not target the poorest households and tend to offer one off payments for adoption of a specific health behavior, such as uptake of a single maternal health service, as opposed to ongoing payments for meeting various conditions [12]. Despite recent growth in popularity for these interventions in SSA few high quality studies have been carried out to evaluate the feasibility or effectiveness of CCTs as a strategy in different SSA contexts, especially relating to uptake of maternal health services [11–13].

In this protocol we describe a mixed methods study in the province of South Kivu, Democratic Republic of Congo (DRC) evaluating the effectiveness of different demand side strategies, including CCTs, to increase maternal health service utilization and the practice of birth spacing. We used an innovative data collection strategy by engaging female community health workers (FCHWs) as part of our survey team. There is limited reporting on FCHWs as data collectors across settings [16–18]. Our study will contribute to the literature exploring demand side interventions while also evaluating contributions of FCHWs as integral members of a research team. Findings on our methodological approach as well as quantitative and qualitative study results will be reported in future publications.

### Context

The DRC – with a population of about 78 million people spread over 2.3 million sq. km – claims one of the highest maternal mortality rates in the world at 846 maternal deaths/100000 live births [19]. After a 2015 mandate the country went from 11 to 26 administrative provinces however South Kivu province where research took place was not affected by this change. Each province is further organized into *zones de santé* (health districts) – geo-political divisions with separate health governing structures under the direction of each corresponding Provincial Department of Health.

The province of South Kivu is located in the eastern region of the DRC, bordering the countries of Burundi and Rwanda. The region erupted in conflict in the wake of the Rwandan genocide in 1994 and ensuing years of civil unrest and regional wars claimed an estimated 5.4 million civilian lives and resulted in millions of internally displaced people (IDP) [20]. The region remains unstable with IDP and refugee camps still in operation and growing, most recently due to political unrest in Burundi. Extremely high rates of sexual violence have come to characterize the region as insecurity stemming from ongoing armed conflicts as well as the largely unsupported reintegration of former members of armed groups into civilian life continues to affect the health, livelihoods and well-being of communities [21].

According to the most recent Demographic and Health Survey 2014 (DHS), most households in South Kivu earn their living in agriculture. Twenty percent of women have no education and the median number of years of education completed by women is 2.7 [19]. 95.8% of South Kivu women received at least one antenatal care (ANC) consultation during their last pregnancy, 92.4% of women reported delivering their last child with a skilled attendant and 7.9% of women in a relationship use a modern method of contraception [19].

The health system in DRC is a tiered system beginning with the most basic health structures offering limited services and increasing in service capacity along a chain of referral. A small number of health posts are located in remote areas offering the most basic medical services. Health posts refer cases, including all births, onto health centers which cover the populations of a specific grouping of villages. Reference health centers are the next level of referral followed by district hospitals and then the provincial reference hospital.

The health system is a fee for service system financed almost entirely by user fees and external donor money. There is very little government financing at the facility level; state money in the form of salaries and consumables that does trickle down to facilities is unreliable and erratic [22]. Transport to facilities, especially in rural, mountainous regions, is scarce and expensive and some health centers are only reachable by 1 h or more on foot. Stock outs continue to be a challenge for some health facilities in the province. Health insurance schemes are available in South Kivu at participating facilities for a fee of $4 USD in rural areas and $7 USD in urban areas per year per person. Subscribers pay a fraction of health service fees at the point of service but in general enrollment is low.

Weak central and local governance spread thin by distance, lack of infrastructure and difficult terrain, varied regional economic development, ongoing conflicts and the patchwork presence of development organizations are probable causes of notable inter- and intra-provincial differences in health outcomes, including maternal health service utilization.For example, the 2014 DHS reports that over 90% of women in South Kivu province delivered their last child with skilled health personnel [19]. In contrast, a 2012 community-based survey of one health zone in South Kivu included in our evaluation reports delivery with skilled health personnel at only 66% [23].

### Study settings

This study takes place in the health districts of Idjwi, Katana and Miti Murhesa. The three districts are situated near the provincial capital of Bukavu, an urban center on the border of Rwanda at the southern tip of Lake Kivu. South Kivu has one of the highest total fertility rates in DRC at 7.7 [19]. The health district of Idjwi is a large island, 40 km in length and 11 km at its widest point [24]. Idjwi is located about 1 h by speed boat from the port of Bukavu and motorized transport on the island is extremely limited especially during the rainy season when roads can quickly become impassable for days at a time. Miti Murhesa and Katana are 1–1.5 h’s drive from Bukavu along varying quality roads, with some villages only reachable by foot.

It is important to note that the three districts have distinct characteristics which could affect health outcomes and will be considered in our final analyses. As an island, the district of Idjwi was spared most of the violence and internal displacement experienced in other health districts. However, logistical challenges of delivering health supplies and motivating health personnel to work on the island are constant challenges to providing care. In addition, agricultural diseases destroying banana and manioc crops significantly changed the economic landscape of the island’s communities as families struggle to feed ever-growing populations from no longer fertile lands. Katana district received heavy humanitarian intervention and aid in the past which could explain comparably better district health indicators at present. Finally, Miti Murhesa district is covered in some places by dense forest where populations within and outside the district seek shelter during periods of ongoing armed conflict. Therefore, the district continues to experience frequent population movements and change.

### Intervention

*Cordaid*, a Dutch non-governmental organization (NGO) with a history of health and development programming in eastern Congo, runs health facility-based, supply-side financing interventions in the zones implicated in our study. Despite expansion of performance-based financing (PBF) to all public health facilities in the implicated health districts NGO program managers noted low uptake of the recommended number of antenatal care (ANC) visits and family planning (FP). Aiming to improve these and other indicators, *Cordaid* implemented a demand-side program in two health districts in South Kivu province to complement ongoing PBF activities. Different demand side strategies are used to incentivize women to access maternal health services and practice birth spacing in two health districts in the study area.

#### Conditional user fee subsidization & conditional cash transfers

In the health district of Idjwi women who attended at least three ANC appointments received a 50% subsidization of user fees for a birth in a public health center or hospital. This *conditional user fee subsidization* covered both uncomplicated and complicated (episiotomy or cesarean section) births. To receive the subsidy women presented a standardized ANC card to the birth facility proving 3 ANC visits during pregnancy within the health district. Unsubsidized facility fees for an uncomplicated birth are officially posted as $10 USD, birth with episiotomy $12 USD and cesarean section births $40 USD but could vary by facility due to facility-specific initiatives and/or informal charges. Fees for medications, additional hospitalization days or electricity surcharges vary between facilities and were not covered by the subsidization program. The most popular insurance scheme in the region, *La Mutuelle,* organized by the Catholic church but open for subscription to anyone, typically pays 20 or 50% of birth fees depending on type of subscription, at participating facilities. Therefore, if a woman participated in the subsidization program and was a member of the health insurance scheme at the 50% level she paid no birth fees at facilities in the insurance scheme network.

In this same health district, beginning 15 months after their last live birth, women could register to participate in a *conditional cash transfer* (CCT) program. Local NGOs registered women and provided community education sessions on the benefits of family planning and birth spacing. Program participants were eligible to receive cash transfers every 3 months that they practiced birth spacing (did not deliver another child) from 15 months up to 27 months after their previous live birth. While women were encouraged to adopt a modern method of contraception, contraception use was not required for program participation.

The amount of the cash transfer increased incrementally with each 3 month period. If a full 27 months of spacing between last live birth and next live birth were respected participants should have received up to $38 USD. Table 1 shows the payment schedule by months of birth spacing. The intervention’s driving principle was the WHO recommendation that women plan at least 24 months in between last live birth and next attempt at conception [25].

**Table 1:** CCT payment schedule by birth spacing interval after last live birth

Birth spacing interval/Months after last birth	Payment to woman
15 months	$3 USD
18 months	$6 USD
21 months	$8 USD
24 months	$9 USD
27 months	$12 USD
Total possible transfer	$38 USD

#### Non-monetary incentives

The second intervention, implemented in the health district of Miti Murhesa, offered women a *non-monetary incentive* (a locally designed and fabricated bracelet) for each ANC appointment they attended for up to three appointments total. Women received a different color bracelet for each ANC appointment.

The health district of Katana served as a comparison group with no demand-side financing interventions implemented by the NGO.

Table 2 describes intervention components by health district.

**Table 2:** Intervention components by health district

Health district	Intervention activities
Idjwi	• 50% subsidization of user fees for birth in public health center or hospital conditional on attendance of at least 3 ANC visits in prenatal period • Cash transfers for every 3 months of birth spacing (not having a subsequent child) beginning 15 months after and up to 27 months after previous live birth
Miti Murhesa	• Non-monetary incentive (locally-made bracelets) given to women for each ANC visit attended for up to three visits
Katana	• Control; no demand side intervention activities

In 2014 the Swiss Tropical and Public Health Institute, a public and global health research and teaching institute associated with the University of Basel in Switzerland, was engaged by *Cordaid* to conduct an evaluation of the comparative effectiveness of each intervention arm and the interventions’ acceptability in the local communities.

### Research aim & Objectives

This study evaluates the effectiveness of non-monetary incentives, conditional user fee subsidization, and CCTs on the utilization of maternal health services including uptake of family planning and their influence on the practice of birth spacing.

Table 3 describes key outcome measures.

**Table 3:** Key outcome measures and their anticipated sources

Outcome	Variable	Longitudinal Survey	Facility Record Review	Semi-structured interviews
Primary Outcomes	ANC in first trimester	X	X	
	ANC completion	X	X	
	Institutional delivery	X	X	
	Birth Interval	X		
Secondary Outcomes	Incidence of unintended pregnancy	X		
	Uptake/use of FP	X		
Exploratory Outcomes	Socio-demographic characteristics of participants/non-participants	X		
	Barriers to service access	X	X	X
	Influence of family members on service use	X		X
	Acceptance of interventions			X
	Identification of intervention mechanisms key to effectiveness			X
	Unintended negative consequences	X	X	X

Specific study objectives were:

1. *To measure the effect of user fee subsidization of facility deliveries conditional on attendance of 3 ANC visits on complete ANC attendance, institutional delivery and post-natal care (PNC).*
2. *To measure the effect of non-monetary incentives linked to attendance of ANC consultations on completion of at least 3 ANC visits and institutional delivery.*
3. *To analyze the comparable effectiveness of conditional user fee subsidization and non-monetary incentives on completion of at least 3 ANC visits and institutional delivery.*
4. *To measure the effectiveness of CCTs on the practice of birth spacing and utilization of contraception (modern and traditional methods).*
5. *To establish profiles of program participants, non-participants and drop-outs and better understand intervention coverage.*
6. *To explore community members’ perceptions and acceptance of program interventions.*

## Methods

### Study design

This study is a quasi-experimental, mixed methods intervention evaluation comprising four main research activities:

1. Formative research phase employing qualitative methods to determine feasibility of planned research activities and inform development of quantitative survey;
2. Community-based, longitudinal survey;
3. Retrospective review of facility records;
4. Qualitative exploration of intervention acceptability and emergent themes, informed by quantitative results.

### Study population

Quantitative survey:

- Women aged 15–49;
- Reside regularly in a household located in one of the randomly selected villages;
- Program participants and non-participants who gave birth from July 2013 – March 2014.

Qualitative (informal interviews, participant observation, in-depth interviews):

- 15 years or older;
- Regularly reside in a household located in one of the randomly selected villages;
- Pregnant or recently delivered women;
- Program participants and non-participants;
- Other community members including husbands/partners, health workers, and community, traditional and religious leaders.

### Sample size and sampling strategy

#### Longitudinal study

Twelve villages were randomly selected using probability proportional to size: 6 from Idjwi, 3 from Katana and 3 from Miti Murhesa. Selection was based on existing health district population data. Each household in each of the 12 villages was approached for permission to identify and recruit women between the ages of 15–49 who regularly reside in the household and who gave birth from July 2013 – March 2014 for participation in the longitudinal survey.

In order to measure a 20% increase in the number of women who practice 24 months of birth spacing from previous live birth to next live birth at 80% power, minimum sample size was set at 645 participants. Provincial ministry and health facility data from 2013 suggested we would need to visit approximately 2500 households on Idjwi (main intervention site) and 2500 households in the comparison areas to identify at least 450 women in the full intervention villages and 450 women in the comparison area villages meeting our eligibility criteria. This sample exceeds our minimum sample and accounts for anticipated 20% non-response and loss to follow up.

#### Retrospective review of facility records

Records of all women attending ANC in the second week of December 2012 (before implementation of the intervention) were taken from ANC registries at each health facility connected to the randomly selected villages from the longitudinal study. The same process was repeated for women attending ANC in the second week of December 2014 (2 years after intervention implementation).

Assuming a prevalence of .5 of at least three ANC visits in the control group a sample of 93 women from each intervention arm allowed detection of 20% difference of completing at least three ANC visits.

#### Qualitative exploration

We conducted in-depth interviews with 40 women from the CCT intervention district and 10 women from the non-monetary incentive district [26–28]. In both intervention groups a purposive sample of program participants and non-participants were recruited. Participants were recruited at health facilities, specifically maternity waiting homes, through FCHWs and then through chain referral in the community.

Health providers and administrators at the village health center, reference health center and general hospital levels were interviewed to gain system perspectives on the interventions as well as facilitators and barriers to women’s use of health services.

Interviews were also conducted with male partners (*n* = 3), older women (*n* = 4) and religious leaders (*n* = 4) to explore specific emergent themes in further detail.

### Ethical approval and consent

This study protocol was approved by the Research Ethics Commission of Northwest and Central Switzerland (EKNZ), the research ethics committee of the Université Catholique de Bukavu (UCB), DRC and the Provincial Ministry of Public Health, South Kivu, DRC.

The study objectives, procedures and participant rights were thoroughly explained to all individuals recruited for participation in longitudinal and qualitative data collection. Signed consent forms were required before data collection began. If participants asked for advice on family planning or other maternal health services they were referred to services already existing in the community including CHWs and facilities where services were available. No participant revealed experiencing sexual violence but the research team was prepared to refer any individuals to the nearest equipped facility should it be necessary. Finally, any participant who revealed experiencing sexual violence will be referred to the nearest facility offering appropriate services. Project money was also available to pay for costs associated with transport to treatment facilities in the case of sexual violence.

### Data collection

The mixed methods design allowed quantitative data to inform the relationships and phenomena to be explored in qualitative collection. In turn, qualitative findings were triangulated with quantitative findings. Inspired by the principles of *grounded theory* [29], qualitative analysis began while data collection was ongoing. This “conversation” between and within quantitative and qualitative data resulted in a more holistic, context-specific exploration and understanding of research topics, including the mechanisms through which the interventions are or are not effective [30].

#### Longitudinal survey

Two FCHWs living in or near each selected village were recruited and trained in basic survey methods. Using paper registers designed specifically for this study, FCHWs visited every household in their assigned village to record the number of women living in a household, their birthdate, date of death (if applicable), the number of children per woman, children’s birthdates and date of death (if applicable).

Field workers were instructed to identify all women who gave birth between July 2013 and March 2014. Female surveyors living outside of the community worked in teams with FCHWs to recruit women who gave birth during target period to respond to a questionnaire. Electronic tablets with the Open Data Kit mobile data collection tool (opendatakit.org) were used to deliver questionnaires. Questionnaires were available in French or Swahili and included questions on socio-demographics, household and individual assets, decision making, maternal health service use, perceptions and uptake of family planning, cultural values and knowledge of/participation in non-monetary incentive or user fee subsidization/CCT programs.

Approximately 6 months after the initial survey round a selection of respondents from Idjwi district was followed up by FCHWs and a local research team member in the community. Using qualitative methods, the teams explored CCT intervention operation, participation and reception in the community.

One year after initial data collection a second round of the quantitative questionnaire was administered to the same participants of the initial survey. Participants were re-identified with help from the FCHWs and data collection emphasized ongoing intervention participation, intervention drop out, uptake of family planning and birth spacing.

#### Retrospective review of facility records

A local field worker visited the health facilities which serve each of the 12 villages in our sample to take pictures of ANC registers from the 2nd week of December 2012. ANC records from the previous 6 months and next 7 months were reviewed to assess each selected woman’s completion of ANC care. Facility birth records were also assessed to determine if the woman delivered in the facility, was referred onto another facility or is not registered as having delivered in a facility. The same procedure was repeated for women attending ANC in the 2nd week of December 2014.

#### Qualitative exploration

Informal interviews with key informants such as government health administrators, health facility staff, CHWs, traditional chiefs, and religious leaders were conducted in the formative stage of research to better understand the socio-cultural landscape, state of maternal health services and service utilization in the study populations. This information informed longitudinal survey design and data collection methods.

After the first round of quantitative survey data collection, an international, French-speaking member of the research team conducted participant observation in several health facilities, maternity waiting homes and communities in the sample population to explore barriers to and decision-making surrounding service use. In-depth, semi-structured interviews with women and other actors followed.

Significant trends observed during analysis of first round survey data and participant observation informed the direction and topics of in-depth interviews. Interviews were ‘in conversation’ with quantitative data by exploring nuances of observed relationships between service (non)-utilization, decision making, cultural values and perceptions of intervention activities.

If participants were not fluent in French, interviews were conducted with the help of a local translator who is fluent in French and the local language of each sample population (Kiswahili, Maashi, and/or Kihavu). Translators were thoroughly trained in confidentiality, qualitative methods and translation techniques.

### Data analysis

#### Quantitative data

Quantitative data will be managed using Excel and the ODK server. STATA v13 (Stata Corp., 2013) will be used during analysis. Chi squared tests will be used to determine associations between categorical variables and logistic regression models and survival analysis will determine predictors of target outcomes. Intervention groups will be compared to the control group and each other to determine intervention effectiveness and differences in outcomes between approaches.

#### Qualitative data

Qualitative data will be translated from the local language into French by the translator during the interview. The research team member conducting the interview will transcribe all interviews in French using f4 transcription software (audiotranskription.de). Atlas.ti v7 will be used to facilitate analysis (atlasti.com).

Analysis followed the principles of grounded theory [29] in that analysis took place over the course of data collection. Emergent themes from initial interviews went on to inform subsequent interviews in a cyclic data collection/analytic process until saturation was reached. Thematic framework analysis using inductive reasoning drove analysis at each stage.

### Results dissemination

The research team will prioritize the dissemination of study results not only to implementing partners and local health authorities but also to the local communities with whom the research was conducted. FCHWs involved in data collection will be approached to help with participatory community dissemination of results after analysis is complete in 2017.

## Discussion

In this protocol we described an evaluation of demand side interventions aiming to increase maternal health service utilization and the practice of birth spacing in a high fertility SSA setting. This approach is innovative in that FCHWs played central roles in research teams’ introduction to communities, understanding of local context and identification of potential participants during data collection. This study will address gaps in several key areas of maternal health research and program implementation in SSA including determinants of maternal health service use and the effectiveness, appropriateness and provider’s/population’s perceptions of different demand side interventions.

The effectiveness, suitability and potential negative consequences of demand side interventions are still highly contested [12]. The growing popularity and enthusiasm surrounding CCTs and other demand side interventions to address specific health outcomes in SSA is not currently matched by a breadth of high quality research [13] and we still have a limited understanding of the ‘black box’ of mechanisms through which CCTs are effective [31]. Some authors question if the conditionality of cash transfers actually prevent the poorest and most vulnerable populations from reaching program benefits and the ethics of dictating how poor individuals should spend their money is a long standing debate [14]. This evaluation aims to fill this research gap, understanding how different demand side approaches including CCTs work as mechanisms for affecting health outcomes in a SSA context and determining who and who is not participating in programs and why. We will also solicit providers’ and community members’ reactions to and perceptions of this program.

In addition, many demand side interventions assume that costs of services are the main barrier to service utilization. While costs are consistently identified in the literature as barriers to maternal health service use and uptake of family planning, evidence also suggests that costs alone are not necessarily the driving factor behind non-uptake. For example, in settings where user fees for maternal health services were abolished up to 50% of women still do not access care [32] and determinants of service use can follow indirect causal pathways [33, 34].The literature 1) emphasizes the importance of prioritizing exploratory and development phases of health programming to better fit interventions to local realities and 2) pushes global health advocates to look beyond cost and understand other factors which could contribute to women’s unwillingness or (in)ability to access services or participate in different programs. These explanatory factors are often hidden in various cultural and/or contextual subtleties; uncovering and understanding them necessitates inventive research strategies, such as the mixed-methods approach with an emphasis on local knowledge presented in this paper. As such, we aim to contextualize and deepen interpretations of quantitative findings through qualitative exploration of lived experiences, perspectives and challenges faced by target populations.

The longitudinal design of our study allows for a comparison of trends between both intervention arms and with the control group. We will be able to identify changes in service utilization and pregnancy status between groups over time and make more robust inferences related to effectiveness of interventions [30]. Qualitative data collection methods and themes will be in conversation with quantitative results as new associations or trends emerge. Recently a number of researchers looking at a variety of health topics in different settings have turned to mixed methods approaches [30, 35–37] in recognition of the importance of understanding the ‘why and how’ of intervention effectiveness and suitability in different contexts [30].

FCHWs are at the core of our data collection teams. FCHWs offer invaluable local knowledge of community norms, local politics and health behaviors, facilitating a positive introduction and launch of our work in study sites. Our approach also provides skills training and financial opportunities for the FCHWs working with us for the duration of the study. FCHWs are now linked with a local research consultancy subcontractor providing human resource support to our study; in future the trained FCHWs could again act as paid surveyors for other community-based studies.

This evaluation partnership reflects a growing recognition of the need to bridge the divide between research and program design and implementation in the field of global health and development. As NGOs and other programming bodies recognize the central importance of rigorous program evaluation in developing successful, culturally-sensitive and cost-effective interventions, research institutions are given the opportunity, challenge and responsibility of translating results and discussions from the academic sphere into real-world application and relevance. This includes prioritization of audience-specific dissemination of results to partners and local populations with whom research was conducted and development of program recommendations which consider real-world constraints faced by NGOs such as implementation logistics, the politics of funding and local socio-politics. It is hoped the research partnership driving this study will serve as an example and encourage funding bodies and other development NGOs to prioritize 1) adequate formative research and programme development phases before implementation goes to scale and 2) rigorous program evaluations, especially those which consider socio-cultural acceptability of intervention components.

It is important to note that the demand side interventions described in this paper take place in the context of an ongoing supply-side, PBF intervention also implemented by the NGO Cordaid in the three health districts included in this study. While interviews with health providers, especially administrators, will undoubtedly touch on PBF our evaluation only concerns outcomes for user fee subsidization and CCTs for birth spacing. Explanations of PBF interventions, evidence for their effectiveness and their possible synergies with CCT programs can be found elsewhere [30, 38, 39] and could be a topic for further research in this context.

A more ideal approach to evaluating intervention activities would have been a mixed methods pre−/post-test study design to measure outcomes before and after intervention implementation. However, the research team was contracted to begin research activities after the start of the intervention making a baseline survey impossible. The mixed methods comparative longitudinal design with control group was chosen by the research team as a feasible alternative for producing robust results which will be helpful in informing the direction of future NGO programming. Despite this necessary compromise in approach, we believe our study in its current design will result in accurate and relevant findings.

## Abbreviations

ANC: Antenatal care
CCTs: Conditional cash transfers
CHW: Community health worker
DHS: Demographic and health survey
DRC: Democratic Republic of Congo
FCHW: Female community health worker
FP: Family planning
IDP: Internally displaced person
NGO: Non-governmental organization
PBF: Performance-based financing
PNC: Post-natal care
SSA: Sub-Saharan Africa
